# Effect of an mHealth self‐help intervention on readmission after adult cardiac surgery: Protocol for a pilot randomized controlled trial

**DOI:** 10.1111/jan.15104

**Published:** 2021-11-28

**Authors:** Rochelle Wynne, Joanne Nolte, Stacey Matthews, Jennifer Angel, Ann Le, Andrew Moore, Tina Campbell, Caleb Ferguson

**Affiliations:** ^1^ Western Sydney Nursing & Midwifery Research Centre Blacktown Clinical & Research School Western Sydney University & Western Sydney Local Health District Blacktown Hospital Blacktown New South Wales Australia; ^2^ School of Nursing & Midwifery Deakin University Geelong Victoria Australia; ^3^ The Royal Melbourne Hospital Parkville Victoria Australia; ^4^ National Heart Foundation Docklands Victoria Australia; ^5^ Liverpool Hospital South West Sydney Local Health District Liverpool New South Wales Australia; ^6^ Healthily Pty Ltd Melbourne Victoria Australia

**Keywords:** cardiac surgery, mHealth, patient participation, randomized controlled trial, readmission, representation

## Abstract

**Aim:**

To describe a protocol for the pilot phase of a trial designed to test the effect of an mHealth intervention on representation and readmission after adult cardiac surgery.

**Design:**

A multisite, parallel group, pilot randomized controlled trial (ethics approval: HREC2020.331‐RMH69278).

**Methods:**

Adult patients scheduled to undergo elective cardiac surgery (coronary artery bypass grafting, valve surgery, or a combination of bypass grafting and valve surgery or aortic surgery) will be recruited from three metropolitan tertiary teaching hospitals. Patients allocated to the control group with receive usual care that is comprised of in‐patient discharge education and local paper‐based written discharge materials. Patients in the intervention group will be provided access to tailored ‘GoShare’ mHealth bundles preoperatively, in a week of hospital discharge and 30 days after surgery. The mHealth bundles are comprised of patient narrative videos, animations and links to reputable resources. Bundles can be accessed via a smartphone, tablet or computer. Bundles are evidence‐based and designed to improve patient self‐efficacy and self‐management behaviours, and to empower people to have a more active role in their healthcare. Computer‐generated permuted block randomization with an allocation ratio of 1:1 will be generated for each site. At the time of consent, and 30, 60 and 90 days after surgery quality of life and level of patient activation will be measured. In addition, rates of representation and readmission to hospital will be tracked and verified via data linkage 1 year after the date of surgery.

**Discussion:**

Interventions using mHealth technologies have proven effectiveness for a range of cardiovascular conditions with limited testing in cardiac surgical populations.

**Impact:**

This study provides an opportunity to improve patient outcome and experience for adults undergoing cardiac surgery by empowering patients as end‐users with strategies for self‐help.

**Trial registration:**

Australian New Zealand Clinical Trials Registry (ANZCTR): ACTRN12621000082808.

## 1 INTRODUCTION

Readmission is multifaceted, but patient driven, potentially avoidable and costly. When readmission rates have been investigated there are consistent features evident in the literature. Literature is generally focused on the efficacy of readmission rates as a quality of care metric (Kansagara et al., 2011), usually in a variety of settings and medical patient populations (Hansen et al., 2011; Leppin et al., 2014). Models have been devised to predict which patients might be readmitted, why patients might require readmission, and if at risk of readmission, which pre‐discharge, post‐discharge or in‐patient interventions might reduce the risk. In the cardiac surgery domain, interest in readmission risk prediction coincided with interest in readmission as a quality‐of‐care metric and there is a plethora of literature from the United States examining readmission risk. In the Australian and European context there is minimal research focused on reasons for or risk of readmission for cardiac surgical candidates.

The utility of social, environmental and system factors that influence patients’ ability to enact self‐care behaviours or access supports to avoid readmission is also under‐investigated. Similarly, the impact of health literacy, patient participation and shared decision‐making on rates of readmission is not well understood. Models are constructed using retrospective administrative data, real‐time administrative data or less frequently prospective primary data collection. In data from the US there is some consistency in factors that increase readmission risk across cardiac settings and samples, but a key feature of models to date is their poor predictive ability. Models predict mortality with reasonable specificity and sensitivity, not readmission. Very few studies assess the influence of variables that are indicative of overall health and function. Frailty, social determinants of health and informal caregiver support are rarely evaluated (Kansagara et al., 2011). In addition, process factors such as the timeliness of post‐discharge follow up, primary‐care coordination and quality of inpatient care are not featured (Gallagher et al., 2020). It is postulated that patient activation, or patients’ capacity to access and enact post‐discharge care crucial for optimal recovery, may influence rates of representation and readmission.

### 1.1 Background

When strategies to reduce readmission rates are explored, interventions are generally in the form of a ‘care bundle’ or explicit actions implemented at a particular time point. Pre‐discharge interventions commonly include patient education, medication reconciliation and scheduling of follow‐up prior to discharge. Post‐discharge interventions include follow‐up phone calls, patient‐activated hotlines, ambulatory care services and home visits (Hansen et al., 2011). There is also a range of bridging interventions designed to impact on transition from in‐patient to primary care. These studies enable us to consider optimal strategies at specific trajectory time points, but systematic review has revealed poor study description, methodological flaws and the use of ‘care bundles’ make it difficult to determine the efficacy of any single intervention on readmission rate reduction (Hansen et al., 2011). Leppin et al. (2014) published a systematic review of interventions for the prevention of readmission that scrutinized features of interventions to determine effect on treatment burden and patients’ capacity to enact burdensome self‐care. These authors concluded that all interventions work to some degree but interventions that support patient capacity for self‐care in the transition from hospital to home are the most effective.

In the cardiac surgery domain, modelling studies have focused on factors that increase risk of readmission and reasons for readmission in American patients with varying levels of readmission risk. Most studies have focused on patients undergoing coronary artery bypass grafts (CABGS). Readmission rates vary widely from 39% in a large multicentre study of patients having surgery in the late 1980s (Steuer et al., 2002) to 6.3% in a low‐risk single centre private sector cohort (Sun et al., 2008). In this relatively homogenous cohort common factors increasing readmission risk are increasing age, female gender, African American race, multiple comorbid conditions and postoperative complications (Hannan et al., 2011; Jarvinen et al., 2003; Vaccarino et al., 2003). Specific studies have also linked additional patient factors including obesity (Rockx et al., 2004), preoperative inflammatory markers (Brown et al., 2013) and diabetes (Stewart et al., 2000) to readmission risk. Few studies have investigated process factors such as ‘off pump’ surgery (Karolak et al., 2007) and ‘fast track’ early discharge (Gooi et al., 2007).

Early discharge has been linked to increased readmission rates into post‐acute care settings and subsequent increased length of stay (LOS) in those settings (Bohmer et al., 2002; Cowper et al., 2007). Acute care facilitates avoid penalty by discharging patients early and admitting them to transitional or sub‐acute care facilities (Lazar et al., 2001). Postoperative LOS is an independent predictor of readmission irrespective of postoperative complications and when LOS in the intensive care unit (ICU) is increased, readmission rates have been reported to be as high as 62% (Lagercrantz et al., 2010). Readmission rates do not differ in studies comparing centres with and without specialist cardiac services (Novick et al., 2007). Very few studies have explored social determinants of health but there has been interest in exploring the effect of mental health on outcome after cardiac surgery, including rates of readmission. Levels of stress, anxiety and depression (Oxlad et al., 2006; Tully et al., 2008) are reliable predictors of poor outcome and increased rates of readmission but are rarely included in models of prediction.

The most common reasons for readmission vary and include arrhythmias 2.4% (Efthymiou & O'Regan, 2011) to 23.1% (Sun et al., 2008), pneumonia or respiratory complications 0.5% (Efthymiou & O'Regan, 2011) to 18%, infection 1.3% (Efthymiou & O'Regan, 2011) to 20% (Cowper et al., 2007) and congestive heart failure 14% (Cowper et al., 2007). Additional reasons reported include constipation, hypotension (Efthymiou & O'Regan, 2011), chest discomfort (Fox et al., 2013), angina and pericardial effusion or tamponade (Sun et al., 2008). In the Australian context Murphy et al. (2008) found living alone was the only independent predictor of readmission in a single centre study of 181 patients of whom 14.4% were readmitted. Another single centre Australian study (Slamowicz et al., 2008) found substantial variability in readmission according to time, where readmission rates were 7% at 7 days, 15.2% at 30 days and 32.3% at 6 months post discharge. In more recent research Fox et al. (2013) found overall readmission was 26.9% when readmission (15%, 95% CI 10.5–13.7) and re‐presentation (11.9%, 95% CI 13.5–16.5) were differentiated. When cohorts include other common types of cardiac surgery, readmission rates rise further. In a multicentre prospective Canadian study of 5158 patients over 10 years, 30‐day readmission rates were 14.9%, 18.3% and 25% for isolated CABGS, isolated valve and combined CABGS and valve surgery, respectively (Iribarne et al., 2014).

Fewer interventions for readmission reduction in cardiac surgery cohorts have been investigated in contrast to those for general medical or surgical patients. An integrative review of preoperative education as a means of reducing readmission found educational materials, methods of needs assessment and specific teaching methods were under‐investigated (Veronovici et al., 2014). Randomized controlled trials of models of care that incorporate a specialist nurse or nurse practitioner have shown no effect in terms of readmission reduction (Kalogianni et al., 2016; Sawatzky et al., 2013), despite reduced rates of anxiety and improvements in health‐related quality of life. Discharge planning involves coordinating care to ensure a quality and safe transition from hospital to home (Bull et al., 2000). Inadequate discharge planning leaves patients ill‐equipped to manage their care after hospitalization (Boughton & Halliday, 2009; Bull et al., 2000) and increases re‐admission rates to hospital following discharge (Phillips et al., 2004; Shepperd et al., 2013). Effective discharge planning after cardiac surgery is crucial given the context of shortening LOS (Cowper et al., 2006) because an increased amount of care previously delivered in hospital is managed by patients and their families in their home environment (Bauer et al., 2009). However, that discharge planning needs to be co‐designed, patient focused and informed by patients’ narrative.

Following cardiac surgery, the trajectory of recovery requires discharge planning to be organized for three distinct phases of rehabilitation: immediate, intermediate and ongoing. In the immediate phase of rehabilitation, the aim of quality discharge planning is a timely discharge from hospital. During the intermediate phase the aim of quality discharge planning is to reduce unplanned re‐admission to hospital. The aim of quality discharge planning for the ongoing phase of rehabilitation is preparation of patients for long‐term self‐management of health (Shepperd et al., 2013). Robust evidence of the impact of patient participation in interventions for readmission reduction after cardiac surgery, is difficult to locate. In a qualitative study of providers and patients, a desire to participate in earlier conversations to allow time to develop plans for treatment and personal preferences, was expressed (Gainer et al., 2017). Patient participation is a quality indicator in healthcare and in the Australian setting ‘Partnering with Consumers’ is one of the eight National Standards for healthcare delivery. Patient participation is one aspect of care in the acute cardiac surgery context that has the potential to influence patient and surgical outcomes yet patients’ ability and willingness to participate is unclear (McTier et al., 2015). A key dimension of the patient quality and safety experience is being discharged from healthcare at the right time with the right plan (McElroy et al., 2016). Effective discharge planning should enhance patient understanding and engagement that in turn influences patients’ ability to participate in accessing and enacting post‐discharge care crucial to avoid readmission. Before we can tailor discharge planning interventions to suit the needs of this patient cohort there needs to be evidence to establish whether improved self‐efficacy enhances self‐management and as such, the capacity to make decisions that reduce the likelihood of representing to hospital or requiring readmission.

## 2 THE STUDY

### 2.1 Aims

The primary aim of this study is to investigate the effect of an mHealth intervention on recovery, as indicated by representation and readmission rates after adult cardiac surgery. Secondary aims are to assess the effect of the mHealth intervention on quality of life, knowledge, skill and confidence for self‐management using the patient activation measure.

### 2.2 Hypothesis

We hypothesize that the mHealth intervention being tested will enhance patients’ self‐efficacy that will in turn reduce the likelihood of them representing to hospital or requiring readmission for recovery challenges that could be self‐managed.

### 2.3 Design and procedure

A multisite, parallel group, pilot randomized controlled trial will be undertaken. A pilot study is necessary to determine sample size requirements for a fully‐fledged trial. The enrolment, intervention and assessment schedule is illustrated in Table 1. Placement on the surgical waiting list and surgical scheduling for each participating site takes place via telephone consultation with a liaison nurse. At the time of referral and placement on the surgical waiting list, verbal consent for participation will be sought and demographic data along with preoperative measures will be entered into a REDCap (Harris et al., 2009) recruitment log. The liaison nurse will contact an independent off‐site research assistant (RA‐1) to confirm participant recruitment via telephone or email. RA‐1 will allocate the patient to intervention or control.

**TABLE 1:** Schedule of enrolment, intervention and outcome assessment

Timepoint	Enrolment	Allocation	Study period				
			Surgery	Assessment			Close‐out
	*−t*_1_	0	*t*_1_	*t*_2_	*t*_3_	*t*_4_	*t*_5_
Enrolment							
Eligibility screen	X						
Informed consent	X						
Allocation		X					
Interventions							
Preoperative bundle		X					
Intermediate bundle			X				
Ongoing bundle				X			
Assessments							
EQ5D‐5L, PAM	X						
EQ5D‐5L, PAM, representation, readmission				X	X	X	
Mortality							X

Abbreviations: EQ5D‐5L, EuroQol short form quality of life assessment tool; PAM, patient activation measure; *t*_1_, days 1–7 after hospital discharge; −*t*_1_, placed on surgical waiting list; *t*_2_, 30 days after surgery; *t*_3_, 60 days after surgery; *t*_4_, 90 days after surgery; *t*_5_, 12 months after surgery.

The liaison nurse will also contact RA‐2, a specialist cardiothoracic clinical nurse, to confirm participant discharge. At 30‐, 60‐ and 90‐days all study participants will be contacted via telephone by RA‐2 to ascertain whether or not they have represented to hospital or have been readmitted to hospital, and if so why. At the time of these calls, postoperative assessment measures will be completed and entered directly into REDCap by RA‐2.

### 2.4 Sample and recruitment

Participants will be recruited from The Royal Melbourne Hospital (RMH) Victoria, Westmead or Liverpool hospitals, in NSW. The two NSW sites service a similar demographic to RMH. The RMH provides services to the western and northern suburbs of Melbourne, extending to Shepparton, Bendigo, Ballarat and Horsham. The RMH provides specialist services including adult congenital cardiac surgery and has an international reputation in valvular surgery and total arterial revascularization. Annually there are approximately 600 cardiac surgery cases performed at this site. The Western Sydney Local Health District (WSLHD) in NSW is the second most populous and has one of the fastest growing populations, expected to increase by almost half a million people by 2036. The catchment includes more than 120 suburbs, over a million residents and the healthcare provided equates to more than $1.8 billion dollars annually. In this district more than 600 cardiothoracic procedures are performed at Westmead hospital annually. Similarly, the South West Sydney Local Health District (SWSLHD) will have over a million residents by 2021, an increase of 21% since 2011. The population is culturally diverse and the district houses some of the poorest communities in NSW. Liverpool Hospital performs between 350 and 400 cardiothoracic procedures annually.

The liaison nurse at each site leads initial contact with patients and placement on elective surgical waiting lists following referral receipt. They explain next steps and processes for preoperative preparation for surgery. They are also the point of contact for patients and families in the lead up to a date being booked for surgery and after the booking is confirmed. Waiting times on elective surgical lists are variable but most cardiac surgery cases are classified as either category 1 or 2 patients, with a median waiting time of 16–19 days in 2017–2018 (AIHW, 2018). At the conclusion of the usual care discussion the liaison nurse will ask elective patients if they are interested in participating in the study using a pre‐prepared script. If patients agree to participate, they will be informed that there will be no difference in the hospital care they receive and that an information form will be sent to them explaining the study. In addition, the nurse will explain that the study involves being randomized to receive a text/email link to GoShare, so they may or may not get the link, and there will be a series of follow‐up phone calls.

Inclusion criteria are any adult (>18 years of age) undergoing elective CABGS, valve surgery, CABGS and valve or aortic surgery, discharged home in 30 days, able to understand spoken English, and they own and use a smartphone, tablet or computer. Exclusion criteria are patients 18 years of age or younger, patients undergoing thoracic, transplant or non‐elective surgery, not discharged in 30 days, not discharged to their home/primary residence, and unable to understand spoken English without an interpreter.

### 2.5 Intervention

The intervention has been designed by Healthily, a nurse led organization that provides a range of health resources for consumers and clinicians focused on building patient knowledge and capacity for self‐help. GoShare Healthcare is Healthily's flagship product and a customized content distribution platform for health professionals to share up‐to‐date, accurate and engaging healthcare resources tailored to the needs of individual patients. An extensive process of consultation and content development is undertaken with clinician and patient involvement to ensure that materials are appropriate, informative and reliable. The cardiac surgery platform contains a patient narrative library with a series of videos describing patient and carer experiences, in addition to animations and links to reputable on‐line resources. Evidence indicates patient narratives have a positive impact on patient education because the information they provide is vivid, engaging, relatable and understandable (Mazor et al., 2007; Winterbottom et al., 2008). When contrasted to written or factual education and communication strategies, narratives are a useful mechanism for encouraging patients to participate in shared decision‐making (Elwyn et al., 2006; Shaffer & Zikmund‐Fisher, 2013). Participants who feature in these videos are positive role models who share their experiences of the day‐to‐day challenges and strategies they use to manage their journey as a patient. The video series is based on current evidence and is designed to improve patient self‐efficacy and self‐management behaviours, and to empower people to play a more active role in their healthcare. Go Share has been developed to support integrated person‐centred care, health literacy and sharing health information. Customized care bundles can be sent to patients via email or SMS on an ad hoc basis or automatically delivered as a digital program at selected times. The platform is currently viewed on over 18,000 screens in over 80 hospitals for specific bundles, not including the cardiac surgery bundle.

The GoShare cardiac surgery bundles have patient narrative videos focused on receiving a diagnosis that requires surgical intervention and preparing for heart surgery, intermediate recovery after heart surgery and ongoing recovery after heart surgery. The videos are delivered by a mixture of male and female patients representing young to older adults. The patients in the videos do not discuss specifics of surgery or medical management, the focus is on strategies for self‐help and self‐efficacy in managing the cardiac surgical experience. The information sheets in the bundles link to resources such as those provided by the Australian Centre for Heart Health and the National Heart Foundation. In the bundle homepage there is an option for patients to provide feedback. To date the cardiac surgery bundle, whilst available for use, has not been tested for effect. Scheduled messaging for the mHealth intervention, as per patient preference via email or SMS, will take place at the time of randomization. The preoperative intervention bundle link will be sent once randomized. On the day of discharge from hospital after surgery, liaison nurses will contact RA‐1 to confirm discharge and trigger distribution of a link to the postoperative immediate recovery bundle in 7 days of discharge. The intermediate recovery bundle link will be sent in 30 days of the day of surgery.

### 2.6 Outcome measures

This prospective multisite pilot study has been designed to test the effect of the cardiac surgery bundle on recovery as measured by representation and readmission rates in the intermediate and ongoing phases of rehabilitation from heart surgery. When readmission rates have been explored, a positive linear relationship with time after surgery is evident (Slamowicz et al., 2008). The primary outcome measures for this trial are representation and readmission in 30, 60, 90 days and at 12 months post surgery (reported separately). Readmission is a proxy for poor patient recovery and rates at 30 days are approximately 14% for isolated CABGS, incrementally increasing for valve, and CABGS with valve, respectively in Australian (Leppin et al., 2014) and international settings (Sun et al., 2008). Recent research emphasis the need to distinguish between representation and readmission that in combination occur in almost 27% of patients (Fox et al., 2013). Secondary outcomes include quality of life as measured by the short form EuroQol (EQ5D‐5L) (Brooks, 1996), and knowledge, skill and confidence for self‐management using the patient activation measure (PAM) (Hibbard et al., 2005). These measures will be assessed at baseline following enrolment and each time the primary outcome is assessed.

Testing in the pilot phase will enable sample size calculation based on the rate of 30‐day readmission to determine adequate power necessary for an ongoing prospective trial. Power analysis will be based on the assumption that readmission rates will be lowest at 30 days. Ongoing interim analyses will be undertaken to assess intervention effect on representation rate that is poorly reported, particularly in the Australian context. To be 95% confident that the readmission rate is in 10%, assuming 15% of the population will be readmitted, a total sample size of 49 patients is required. Loss to follow up, as indicated by 30‐day outcomes captured in the Australian and New Zealand Cardiac and Thoracic Surgeons (ANZSCTS) Database Registry, is approximately 4% annually. Each site performs an average of 10–15 cases per week. The aim is to recruit 20 patients from each site to a total of 60 patients, allowing for loss to follow up, prior to interim analyses in this pilot study. If two patients per week from each site can be recruited the trial should take approximately 10–12 weeks.

RA‐1 will maintain an electronic randomization schedule, and using a computer‐generated permuted block randomization schedule, randomize the patient to intervention or control. The enrolment, randomization and follow‐up schedules will be housed in separate sheets in REDCap to ensure liaison nurses and RA‐2 are blinded to allocation. It is not possible to blind patients to the intervention, and they may disclose allocation to RA‐2. Data analyses will be complete by an investigator not involved in recruitment, allocation or outcome measurement.

### 2.7 Data storage and analysis

REDCap (Harris et al., 2009) is a secure web application used exclusively to support data capture for research studies. The intuitive interface provides concurrent data validation with entry, 256 bit encryption between the data entry client and the server, secure access, an audit trail for tracking data manipulation and export procedures, automated export procedures for data downloads to common statistical packages, data import from external sources and advanced features such as branching logic, calculated fields and data quality checks. Developed and maintained by a team at the Vanderbuilt University it is licensed free of charge by Melbourne Health (MH). The MH copy of the REDCap application and data are housed in MH secure encrypted servers. The data are securely backed up off site as per standard procedures for data security and local support is provided by MH. The study database will be created in REDCap hosted by MH. As per the approved protocol, study participants will be allocated a unique study code and identifiers entered into the study database that is configured to flag these as such in REDCap, which then provides additional protection for these data elements. Identifiers will only be available to specified individuals on the project. Identifiers will be automatically removed from all printed material generated by REDCap where the user does not have permission to access identifiers. The principal investigator (PI) will be the only member of the project team able to download data from REDCap.

The downloaded master file for statistical analyses will contain a unique Code ID so data will be reidentifiable. Data will be exported from REDCap into IBM SPSS Statistics for Windows (version 27; IBM Corp.) for analysis. Preoperative, intraoperative and perioperative data will be extracted from the ANZSCTS repository following 90‐day follow up. A minimum of 12 months after the date of surgery for the last enrolled patient, ANZSCTS data linked to the National Death Index will be accessed for the study cohort and combined with existing data that will then be screened for inconsistencies and errors. On completion of cleaning, linking variables will be removed, data will become non‐identifiable and aggregate analyses will be undertaken. Categorical data will be presented as frequency and proportion and continuous data as mean and standard deviation where normally distributed, or median with quartiles.

Preoperative demographic data, operative data and early postoperative complications will be compared between intervention and control group. Frequency of representation, readmission and mHealth metrics will be reported as proportions and the EQ5D‐5L and PAM endpoints are scale measures that will also be compared between intervention and control groups. The approach to analysis of repeated measures results from the EQ5D‐5L and PAM will be dependent on loss to follow up and normality of distribution. Categorical variables will be analysed using chi‐square (χ^2^) or two‐tailed Fisher's exact test with appropriate degrees of freedom to test for equality of proportions. Independent samples *t*‐tests (two‐tailed) will be used to test for equality of means for continuous variables or the non‐parametric equivalent Kruskal–Wallis test. Univariate factors with a *p* value ≤0.25 will be considered eligible for multivariate analysis. Multivariate logistic regression will be used to identify predictors of readmission. Direct entry techniques will be contrasted with backward stepwise selection to build a parsimonious explanatory model of association. Model sensitivity and specificity will be reviewed using receiver operating characteristic curve analysis with 95% confidence intervals calculated.

### 2.8 Ethical considerations

This trial has ethics approval under the auspices of national mutual acceptance from the Melbourne Health Human Ethics and Research Committee (HREC2020.331‐RMH69278). The trial poses low or minimal risk to participants and verbal informed consent has been approved in the context of COVID‐19 restrictions and a concomitant reduction in preoperative face‐to‐face appointments. Patients will be agreeing to an opportunity to access GoShare materials and receive the follow‐up phone calls from an expert cardiothoracic nurse (RA‐2). GoShare access further implies patients’ agreement. At the time of each follow‐up phone call RA‐2 will again confirm that the patient agrees to answers questions about their quality of life and level of activation in healthcare.

The point at which the greatest patient burden or risk of discomfort is anticipated during the follow‐up phone calls. To counteract this risk an expert cardiothoracic nurse will conduct these calls. Should there be any concerns raised that warrant further investigation RA‐2 is well placed to provide appropriate advice to patients that may need to referral to support services. Liaison nurses, RA‐2 and the study PI will have monthly team meetings to ensure streamlined processes are in place and to ensure concerns are effectively addressed. If a patient chooses to withdraw from the study, data collected to date will be non‐identifiable and retained for the purposes of aggregate analyses.

## 3 DISCUSSION

Data from the Independent Hospital Pricing Authority indicates that in 2017 the average cost of a patient re‐presenting to an Australian Emergency Department was $533 and, if subsequently admitted, $969. In 2019 there were over 15,000 adult cardiac surgery procedures performed in Australia. Readmission was on average 10.2% for isolated CABG patients, 10.4% for isolated aortic valve surgery and 10.9% for combined CABG and valve. In 2017 the average rate of readmission for all case types was approximately 14% (Tran et al., 2019). This equates to an approximate national spend of between $1.04 and $1.9 million for the year. NSW, the most populous Australian state, undertakes 30% of cases annually (Tran et al., 2019) thus there is a minimum spend of $336,000 for re‐presentation and $610,000 for readmission, before factoring in the cost of subsequent hospitalization, if that is required, in NSW alone. The national Australian annual cost of readmission based on an average rate of 14% and 2017 pricing would be in excess of $3.1 million. The true incidence of re‐presentation and readmission in the year after surgery is however, unknown. Rudimentary financial estimates fail to consider impact on patient flow, health service efficiency and effectiveness, or the patient and family experience.

COVID‐19 has created extraordinary circumstances that will persist for some time. Despite the plethora of negative outcomes associated with the pandemic there have been some positive benefits for patients, one of which is improved access to telehealth (Wynne, Conway, et al., 2021; Wynne, Davidson, et al., 2021). Interventions delivered using smartphone or mHealth technologies have proven effectiveness for a range of cardiovascular conditions under normal circumstances (Gallagher et al., 2020) and abnormal circumstances (Martorella et al., 2021). It is not clear what role patients have in influencing rates of readmission after cardiac surgery. This trial will provide evidence to substantiate the rate of representation and readmission and the effect of an intervention designed to empower patients to actively participate in their own care, on reduction of these rates. In addition, findings from this research will identify predictors of representation or readmission specific to the Australian context.

### 3.1 Limitations

This pilot study is to our knowledge, the first to test the effect of an mHealth intervention on recovery after adult cardiac surgery. The feasibility of the intervention has not previously been tested in a cardiac surgical context. In a pilot study for weight management in young adults the GoShare platform was shown to be a feasible and acceptable mechanism to augment self‐efficacy and self‐management but no more effective than usual care (Hebden et al., 2014). However, there was variability between groups in this study of 70 patients and the authors concluded a larger sample size was needed. Interim analyses in this pilot will enable timely assessment of effect to inform ongoing recruitment.

An additional limitation is the ongoing impact of the COVID‐19 pandemic on elective surgery and the patient journey. When patients with cardiovascular disease requiring surgery do get an elective booking, they are frequently having to traverse the process in isolation making the capacity for self‐management more important than ever. Health services have been drastically reduced with preadmission clinics often closed or limited, the availability of telehealth variable and accompanying carers or next of kin banned. Almost 50% of cardiac rehabilitation services have ceased (Inglis et al., 2020). A resource intensive specialty, cardiac surgery is reliant on the availability of ICU beds for immediate recovery. To date, elective surgery cancellation is a common strategy employed to optimize ICU bed availability. Whilst it can be argued that nothing in cardiac surgery is truly elective, the specialty is not immune from this approach (Wynne & Smith, 2021). An additional consideration is the ongoing burden of care on critical care nurses during the pandemic that also impacts on ICU bed availability (Wynne, Conway, et al., 2021; Wynne, Davidson, et al., 2021). These factors in combination, will influence the capacity for elective surgery and as such, recruitment into the trial.

A final study limitation is the inability to blind patients from being in the intervention group that should be counteracted by the process of random selection and allocation by an investigator (RA‐1) not involved in follow‐up and the collection of outcome measures. Patients who are allocated to the intervention may reveal that to the investigator (RA‐2) collecting outcomes but valid, reliable objective instruments are being used to capture outcomes that should eliminate any risk of response bias.

## 4 CONCLUSIONS

Representation and readmission after cardiac surgery are potentially avoidable and costly. Interventions using mHealth technologies have demonstrated effectiveness for a range of cardiovascular conditions with limited evaluation in cardiac surgical populations. Findings from this study will establish whether improved self‐efficacy enhances self‐management and as such, the capacity to make decisions that reduce the likelihood of representing to hospital or requiring readmission. Patient participation and experience during the trajectory of recovery from cardiac surgery are neglected aspects of research in this specialty. This study will provide evidence to substantiate the value of mHealth and self‐efficacy for self‐management in patients undergoing cardiac surgery. Findings will also provide longitudinal representation and readmission data for quality benchmarking. A model of prediction will be built to inform preoperative risk assessment and discharge planning processes. In combination, outcomes from this research will provide a platform from which a program of research focused on future interventional studies augmenting patient patient‐led recovery can be developed.

## CONFLICT OF INTEREST

No conflict of interest has been declared by the author(s).

## AUTHOR CONTRIBUTIONS

All authors have agreed on the final version and meet at least one of the following criteria (recommended by the ICMJE: http://www.icmje.org/recommendations/):

1. Substantial contributions to conception and design, acquisition of data or analysis and interpretation of data;
2. Drafting the article or revising it critically for important intellectual content.

### PEER REVIEW

The peer review history for this article is available at https://publons.com/publon/10.1111/jan.15104.

## Data Availability

On completion of the trial the data that support the findings of this study will be available on request from the corresponding author. The data will not be publicly available due to ethical restrictions.

## References

[jan15104-bib-0001] Australian Institute of Health & Welfare . (2018 ). Elective surgery waiting times 2017‐2018: Australian hospital statistics (Health services series, Issue. A). AIHW.

[jan15104-bib-0002] Bauer, M. , Fitzgerald, L. , Haesler, E. , & Manfrin, M. (2009). Hospital discharge planning for frailolder people and their family. Are we delivering best practice? A review of the evidence. Journal of Clinical Nursing, 18(18), 2539–2546. 10.1111/j.1365-2702.2008.02685.x 19374695

[jan15104-bib-0003] Bohmer, R. M. , Newell, J. , & Torchiana, D. F. (2002). The effect of decreasing length of stay on discharge destination and readmission after coronary bypass operation. Surgery, 132(1), 10–15. 10.1067/msy.2002.125358 12110788

[jan15104-bib-0004] Boughton, M. , & Halliday, L. (2009). Home alone: Patient and carer uncertainty surrounding discharge with continuing clinical care needs. Contemporary Nurse, 33(1), 30–40. 10.5172/conu.33.1.30 19715493

[jan15104-bib-0005] Brooks, R. (1996). EuroQol: The current state of play. Health Policy, 37(1), 53–72. 10.1016/0168-8510(96)00822-6 10158943

[jan15104-bib-0006] Brown, J. R. , Landis, R. C. , Chaisson, K. , Ross, C. S. , Dacey, L. J. , Boss, R. A. Jr , Helm, R. E. , Horton, S. R. , Hofmaster, P. , Jones, C. , Desaulniers, H. , Westbrook, B. M. , Duquette, D. , Leblond, K. , Quinn, R. D. , Magnus, P. C. , Malenka, D. J. , & Discipio, A. W. (2013). Preoperative white blood cell count and risk of 30‐day readmission after cardiac surgery. International Journal of Inflammation, 2013, 781024. 10.1155/2013/781024 23970996 PMC3732591

[jan15104-bib-0007] Bull, M. J. , Hansen, H. E. , & Gross, C. R. (2000). A professional‐patient partnership model of discharge planning with elders hospitalized with heart failure. Applied Nursing Research, 13(1), 19–28. 10.1016/s0897-1897(00)80015-4 10701280

[jan15104-bib-0008] Cowper, P. A. , DeLong, E. R. , Hannan, E. L. , Muhlbaier, L. H. , Lytle, B. L. , Jones, R. H. , Holman, W. L. , Pokorny, J. J. , Stafford, J. A. , Mark, D. B. , & Peterson, E. D. (2006). Trends in postoperative length of stay after bypass surgery. American Heart Journal, 152(6), 1194–1200. 10.1016/j.ahj.2006.07.017 17161075

[jan15104-bib-0009] Cowper, P. A. , DeLong, E. R. , Hannan, E. L. , Muhlbaier, L. H. , Lytle, B. L. , Jones, R. H. , Holman, W. L. , Pokorny, J. J. , Stafford, J. A. , Mark, D. B. , & Peterson, E. D. (2007). Is early too early? Effect of shorter stays after bypass surgery. Annals of Thoracic Surgery, 83(1), 100–107. 10.1016/j.athoracsur.2006.08.001 17184638

[jan15104-bib-0010] Efthymiou, C. A. , & O'Regan, D. J. (2011). Postdischarge complications: What exactly happens when the patient goes home? Interactive Cardiovascular and Thoracic Surgery, 12(2), 130–134. 10.1510/icvts.2010.249474 21123196

[jan15104-bib-0011] Elwyn, G. , O'Connor, A. , Stacey, D. , Volk, R. , Edwards, A. , Coulter, A. , Thomson, R. , Barratt, A. , Barry, M. , Bernstein, S. , Butow, P. , Clarke, A. , Entwistle, V. , Feldman‐Stewart, D. , Holmes‐Rovner, M. , Llewellyn‐Thomas, H. , Moumjid, N. , Mulley, A. , Ruland, C. , … Whelan, T. (2006). Developing a quality criteria framework for patient decision aids: online international Delphi consensus process. BMJ, 333(7565), 417. 10.1136/bmj.38926.629329.AE 16908462 PMC1553508

[jan15104-bib-0012] Fox, J. P. , Suter, L. G. , Wang, K. , Wang, Y. , Krumholz, H. M. , & Ross, J. S. (2013). Hospital‐based, acute care use among patients within 30 days of discharge after coronary artery bypass surgery. Annals of Thoracic Surgery, 96(1), 96–104. 10.1016/j.athoracsur.2013.03.091 23702228 PMC3758868

[jan15104-bib-0013] Gainer, R. A. , Curran, J. , Buth, K. J. , David, J. G. , Legare, J. F. , & Hirsch, G. M. (2017). Toward optimal decision making among vulnerable patients referred for cardiac surgery: A qualitative analysis of patient and provider perspectives. Medical Decision Making, 37(5), 600–610. 10.1177/0272989X16675338 27803362

[jan15104-bib-0014] Gallagher, R. , Parker, H. , Zhang, L. , Kirkness, A. , Roach, K. , Belshaw, J. , Glinatsis, H. , Gallagher, P. , & Neubeck, L. (2020). Target audience and preferences related to an Australian Coronary Heart Disease Specific Mobile App: A mixed methods study. Heart, Lung & Circulation, 29(5), 696–702. 10.1016/j.hlc.2019.05.178 31235365

[jan15104-bib-0015] Gooi, J. , Marasco, S. , Rowland, M. , Esmore, D. , Negri, J. , & Pick, A. (2007). Fast‐track cardiac surgery: application in an Australian setting. Asian Cardiovascular and Thoracic Annals, 15(2), 139–143. 10.1177/021849230701500212 17387197

[jan15104-bib-0016] Hannan, E. L. , Zhong, Y. , Lahey, S. J. , Culliford, A. T. , Gold, J. P. , Smith, C. R. , Higgins, R. S. , Jordan, D. , & Wechsler, A. (2011). 30‐day readmissions after coronary artery bypass graft surgery in New York State. JACC: Cardiovascular Interventions, 4(5), 569–576. 10.1016/j.jcin.2011.01.010 21596331

[jan15104-bib-0017] Hansen, L. O. , Young, R. S. , Hinami, K. , Leung, A. , & Williams, M. V. (2011). Interventions to reduce 30‐day rehospitalization: A systematic review. Annals of Internal Medicine, 155(8), 520–528. 10.7326/0003-4819-155-8-201110180-00008 22007045

[jan15104-bib-0018] Harris, P. A. , Taylor, R. , Thielke, R. , Payne, J. , Gonzalez, N. , & Conde, J. G. (2009). Research electronic data capture (REDCap)–a metadata‐driven methodology and workflow process for providing translational research informatics support. Journal of Biomedical Informatics, 42(2), 377–381. 10.1016/j.jbi.2008.08.010 18929686 PMC2700030

[jan15104-bib-0019] Hebden, L. , Cook, A. , van der Ploeg, H. P. , King, L. , Bauman, A. , & Allman‐Farinelli, M. (2014). A mobile health intervention for weight management among young adults: a pilot randomised controlled trial. Journal of Human Nutrition & Dietetics, 27(4), 322–332. 10.1111/jhn.12155 23992038

[jan15104-bib-0020] Hibbard, J. H. , Mahoney, E. R. , Stockard, J. , & Tusler, M. (2005). Development and testing of a short form of the patient activation measure. Health Services Research, 40(6 Pt 1), 1918–1930. 10.1111/j.1475-6773.2005.00438.x 16336556 PMC1361231

[jan15104-bib-0021] Inglis, S. C. , Naismith, C. , White, K. , Hendriks, J. M. , Bray, J. , Hickman, L. D. , Aldridge, C. , Bardsley, K. , Cameron, J. , Candelaria, D. , Cartledge, S. , Du, H. , Ferguson, C. , Martin, L. , Selkow, T. , Xu, X. , Wynne, R. , Driscoll, A. , Gallagher, R. , … Davidson, P. M. (2020). CSANZ COVID‐19 cardiovascular nursing care consensus statement: Executive summary. Heart, Lung & Circulation, 29(9), 1263–1267. 10.1016/j.hlc.2020.08.001 PMC7413197 32859539

[jan15104-bib-0022] Iribarne, A. , Chang, H. , Alexander, J. H. , Gillinov, A. M. , Moquete, E. , Puskas, J. D. , Bagiella, E. , Acker, M. A. , Mayer, M. L. , Ferguson, T. B. , Burks, S. , Perrault, L. P. , Welsh, S. , Johnston, K. C. , Murphy, M. , DeRose, J. J. , Neill, A. , Dobrev, E. , Baio, K. T. , … O'Gara, P. T. (2014). Readmissions after cardiac surgery: Experience of the National Institutes of Health/Canadian Institutes of Health research cardiothoracic surgical trials network. Annals of Thoracic Surgery, 98(4), 1274–1280. 10.1016/j.athoracsur.2014.06.059 25173721 PMC4186890

[jan15104-bib-0023] Jarvinen, O. , Huhtala, H. , Laurikka, J. , & Tarkka, M. R. (2003). Higher age predicts adverse outcome and readmission after coronary artery bypass grafting. World Journal of Surgery, 27(12), 1317–1322. 10.1007/s00268-003-7033-5 14716500

[jan15104-bib-0024] Kalogianni, A. , Almpani, P. , Vastardis, L. , Baltopoulos, G. , Charitos, C. , & Brokalaki, H. (2016). Can nurse‐led preoperative education reduce anxiety and postoperative complications of patients undergoing cardiac surgery? European Journal of Cardiovascular Nursing, 15(6), 447–458. 10.1177/1474515115602678 26304701

[jan15104-bib-0025] Kansagara, D. , Englander, H. , Salanitro, A. , Kagen, D. , Theobald, C. , Freeman, M. , & Kripalani, S. (2011). Risk prediction models for hospital readmission: A systematic review. JAMA, 306(15), 1688–1698. 10.1001/jama.2011.1515 22009101 PMC3603349

[jan15104-bib-0026] Karolak, W. , Hirsch, G. , Buth, K. , & Legare, J. F. (2007). Medium‐term outcomes of coronary artery bypass graft surgery on pump versus off pump: Results from a randomized controlled trial. American Heart Journal, 153(4), 689–695. 10.1016/j.ahj.2007.01.033 17383313

[jan15104-bib-0027] Lagercrantz, E. , Lindblom, D. , & Sartipy, U. (2010). Survival and quality of life in cardiac surgery patients with prolonged intensive care. Annals of Thoracic Surgery, 89(2), 490–495. 10.1016/j.athoracsur.2009.09.073 20103328

[jan15104-bib-0028] Lazar, H. L. , Fitzgerald, C. A. , Ahmad, T. , Bao, Y. , Colton, T. , Shapira, O. M. , & Shemin, R. J. (2001). Early discharge after coronary artery bypass graft surgery: Are patients really going home earlier? Journal of Thoracic and Cardiovascular Surgery, 121(5), 943–950. 10.1067/mtc.2001.113751 11326238

[jan15104-bib-0029] Leppin, A. L. , Gionfriddo, M. R. , Kessler, M. , Brito, J. P. , Mair, F. S. , Gallacher, K. , Wang, Z. , Erwin, P. J. , Sylvester, T. , Boehmer, K. , Ting, H. H. , Murad, M. H. , Shippee, N. D. , & Montori, V. M. (2014). Preventing 30‐day hospital readmissions: A systematic review and meta‐analysis of randomized trials. JAMA Internal Medicine, 174(7), 1095–1107. 10.1001/jamainternmed.2014.1608 24820131 PMC4249925

[jan15104-bib-0030] Martorella, G. , Fredericks, S. , Sanders, J. , & Wynne, R. (2021). Breaking pandemic chain reactions: Telehealth psychosocial support in cardiovascular disease during COVID‐19. European Journal of Cardiovascular Nursing, 20(1), 1–2. 10.1093/eurjcn/zvaa011 33570589 PMC7799113

[jan15104-bib-0031] Mazor, K. M. , Baril, J. , Dugan, E. , Spencer, F. , Burgwinkle, P. , & Gurwitz, J. H. (2007). Patient education about anticoagulant medication: Is narrative evidence or statistical evidence more effective? Patient Education and Counseling, 69(1–3), 145–157. 10.1016/j.pec.2007.08.010 17942268

[jan15104-bib-0032] McElroy, I. , Sareh, S. , Zhu, A. , Miranda, G. , Wu, H. , Nguyen, M. , Shemin, R. , & Benharash, P. (2016). Use of digital health kits to reduce readmission after cardiac surgery. Journal of Surgical Research, 204(1), 1–7. 10.1016/j.jss.2016.04.028 27451860

[jan15104-bib-0033] McTier, L. , Botti, M. , & Duke, M. (2015). Patient participation in medication safety during an acute care admission. Health Expectations, 18(5), 1744–1756. 10.1111/hex.12167 24341439 PMC5060834

[jan15104-bib-0034] Murphy, B. M. , Elliott, P. C. , Le Grande, M. R. , Higgins, R. O. , Ernest, C. S. , Goble, A. J. , Tatoulis, J. , & Worcester, M. U. (2008). Living alone predicts 30‐day hospital readmission after coronary artery bypass graft surgery. European Journal of Cardiovascular Prevention and Rehabilitation, 15(2), 210–215. 10.1097/HJR.0b013e3282f2dc4e 18391650

[jan15104-bib-0035] Novick, R. J. , Fox, S. A. , Stitt, L. W. , Butler, R. , Kroh, M. , Hurlock‐Chorostecki, C. , Harris, C. , & Cheng, D. C. (2007). Impact of the opening of a specialized cardiac surgery recovery unit on postoperative outcomes in an academic health sciences centre. Canadian Journal of Anaesthesia, 54(9), 737–743. 10.1007/BF03026870 17766741

[jan15104-bib-0036] Oxlad, M. , Stubberfield, J. , Stuklis, R. , Edwards, J. , & Wade, T. D. (2006). Psychological risk factors for cardiac‐related hospital readmission within 6 months of coronary artery bypass graft surgery. Journal of Psychosomatic Research, 61(6), 775–781. 10.1016/j.jpsychores.2006.09.008 17141665

[jan15104-bib-0037] Phillips, C. O. , Wright, S. M. , Kern, D. E. , Singa, R. M. , Shepperd, S. , & Rubin, H. R. (2004). Comprehensive discharge planning with postdischarge support for older patients with congestive heart failure: A meta‐analysis. JAMA, 291(11), 1358–1367. 10.1001/jama.291.11.1358 15026403

[jan15104-bib-0038] Rockx, M. A. , Fox, S. A. , Stitt, L. W. , Lehnhardt, K. R. , McKenzie, F. N. , Quantz, M. A. , Menkis, A. H. , & Novick, R. J. (2004). Is obesity a predictor of mortality, morbidity and readmission after cardiac surgery? Canadian Journal of Surgery, 47(1), 34–38. https://www.ncbi.nlm.nih.gov/pubmed/14997923 PMC3211805 14997923

[jan15104-bib-0039] Sawatzky, J. A. , Christie, S. , & Singal, R. K. (2013). Exploring outcomes of a nurse practitioner‐managed cardiac surgery follow‐up intervention: a randomized trial. Journal of Advanced Nursing, 69(9), 2076–2087. 10.1111/jan.12075 23317314

[jan15104-bib-0040] Shaffer, V. A. , & Zikmund‐Fisher, B. J. (2013). All stories are not alike: a purpose‐, content‐, and valence‐based taxonomy of patient narratives in decision aids. Medical Decision Making, 33(1), 4–13. 10.1177/0272989x12463266 23065418

[jan15104-bib-0041] Shepperd, S. , Lannin, N. A. , Clemson, L. M. , McCluskey, A. , Cameron, I. D. , & Barras, S. L. (2013). Discharge planning from hospital to home. Cochrane Database of Systematic Review, 1, CD000313. 10.1002/14651858.CD000313.pub4 23440778

[jan15104-bib-0042] Slamowicz, R. , Erbas, B. , Sundararajan, V. , & Dharmage, S. (2008). Predictors of readmission after elective coronary artery bypass graft surgery. Australian Health Review, 32(4), 677–683. 10.1071/ah080677 18980563

[jan15104-bib-0043] Steuer, J. , Blomqvist, P. , Granath, F. , Rydh, B. , Ekbom, A. , de Faire, U. , & Stahle, E. (2002). Hospital readmission after coronary artery bypass grafting: Are women doing worse? Annals of Thoracic Surgery, 73(5), 1380–1386. 10.1016/s0003-4975(02)03467-7 12022521

[jan15104-bib-0044] Stewart, R. D. , Campos, C. T. , Jennings, B. , Lollis, S. S. , Levitsky, S. , & Lahey, S. J. (2000). Predictors of 30‐day hospital readmission after coronary artery bypass. Annals of Thoracic Surgery, 70(1), 169–174. 10.1016/s0003-4975(00)01386-2 10921703

[jan15104-bib-0045] Sun, X. , Zhang, L. , Lowery, R. , Petro, K. R. , Hill, P. C. , Haile, E. , Garcia, J. M. , Bafi, A. S. , Boyce, S. W. , & Corso, P. J. (2008). Early readmission of low‐risk patients after coronary surgery. The Heart Surgery Forum, 11(6), E327–E332. 10.1532/HSF98.20071192 19073528

[jan15104-bib-0046] Tran, L. , Williams‐Spence, J. , Shardey, G. C. , Smith, J. A. , & Reid, C. M. (2019). The Australian and New Zealand Society of Cardiac and Thoracic Surgeons Database Program ‐ Two decades of quality assurance data. Heart, Lung & Circulation, 28(10), 1459–1462. 10.1016/j.hlc.2019.03.002 30962063

[jan15104-bib-0047] Tully, P. J. , Baker, R. A. , Turnbull, D. , & Winefield, H. (2008). The role of depression and anxiety symptoms in hospital readmissions after cardiac surgery. Journal of Behavioral Medicine, 31(4), 281–290. 10.1007/s10865-008-9153-8 18398676

[jan15104-bib-0048] Vaccarino, V. , Lin, Z. Q. , Kasl, S. V. , Mattera, J. A. , Roumanis, S. A. , Abramson, J. L. , & Krumholz, H. M. (2003). Sex differences in health status after coronary artery bypass surgery. Circulation, 108(21), 2642–2647. 10.1161/01.CIR.0000097117.28614.D8 14597590

[jan15104-bib-0049] Veronovici, N. R. , Lasiuk, G. C. , Rempel, G. R. , & Norris, C. M. (2014). Discharge education to promote self‐management following cardiovascular surgery: An integrative review. European Journal of Cardiovascular Nursing, 13(1), 22–31. 10.1177/1474515113504863 24042728

[jan15104-bib-0050] Winterbottom, A. , Bekker, H. L. , Conner, M. , & Mooney, A. (2008). Does narrative information bias individual's decision making? A systematic review. Social Science & Medicine, 67(12), 2079–2088. 10.1016/j.socscimed.2008.09.037 18951673

[jan15104-bib-0051] Wynne, R. , Conway, A. , & Davidson, P. M. (2021). Ensuring COVID‐related innovation is sustained. Journal of Advanced Nursing, 77(6), e4–e6. 10.1111/jan.14837 33769571 PMC8251018

[jan15104-bib-0052] Wynne, R. , Davidson, P. M. , Duffield, C. , Jackson, D. , & Ferguson, C. (2021). Workforce management and patient outcomes in the intensive care unit during the COVID‐19 pandemic and beyond: A discursive paper. Journal of Clinical Nursing, 10.1111/jocn.15916 PMC8447459 34184349

[jan15104-bib-0053] Wynne, R. , & Smith, J. A. (2021). Cardiac surgery in Australia during the COVID‐19 global pandemic. Heart, Lung & Circulation, 30(12), 1800–1804. 10.1016/j.hlc.2021.09.008 PMC8479417 34598887

